# Periodicity Intensity of the 24 h Circadian Rhythm in Newborn Calves Show Indicators of Herd Welfare

**DOI:** 10.3390/s22155843

**Published:** 2022-08-04

**Authors:** Victoria Rhodes, Maureen Maguire, Meghana Shetty, Conor McAloon, Alan F. Smeaton

**Affiliations:** 1 School of Veterinary Medicine, University College Dublin, Belfield, D04 V1W8 Dublin, Ireland; 2 School of Computing, Dublin City University, Glasnevin, Dublin 9, Ireland; 3 Insight Centre for Data Analytics, Dublin City University, Glasnevin, Dublin 9, Ireland

**Keywords:** animal welfare, activity monitoring, wearable sensors, data analytics, chronobiology, periodicity

## Abstract

Circadian rhythms are a process of the sleep-wake cycle that regulates the physical, mental and behavioural changes in all living beings with a period of roughly 24 h. Wearable accelerometers are typically used in livestock applications to record animal movement from which we can estimate the activity type. Here, we use the overall movement recorded by accelerometers worn on the necks of newborn calves for a period of 8 weeks. From the movement data, we calculate 24 h periodicity intensities corresponding to circadian rhythms, from a 7-day window that slides through up to 8-weeks of data logging. The strength or intensity of the 24 h periodicity is computed at intervals as the calves become older, which is an indicator of individual calf welfare. We observe that the intensities of these 24 h periodicities for individual calves, derived from movement data, increase and decrease synchronously in a herd of 19 calves. Our results show that external factors affecting the welfare of the herd can be observed by processing and visualising movement data in this way and our method reveals insights that are not observable from movement data alone.

## 1. Introduction

Circadian rhythms play a crucial role in the lives of all living things and are vital for regulating sleep/wake cycles and feeding patterns. A regular circadian rhythm can be linked to good health, wellbeing and a strong immune system. Changes in circadian rhythm are known to occur under stressful conditions and thus an indicator of the intensity of the 24 h circadian rhythm may be a useful indicator of animal welfare.

The regularity of the sleep-wake cycle can be measured using periodicity, the phenomenon whereby an event or behaviour occurs at regular intervals over time. Periodicity identifies patterns in time series data that occur with regular periodic intervals. The detection of periodicity in time series can be calculated using a variety of techniques which were summarised in an extensive literature survey in [1]. In our work, we use power spectral density (PSD) [2] as this allowed us to determine the strength of repeating signals at various time frequencies, though in the end, we focused on just the signal at the 24 h frequency, i.e., the circadian rhythm.

For our study, we used 24 h periodicity intensity, the strength of the periodicity in sensor data at a frequency of 24 h, calculated within a sliding time window. A strong periodicity indicates behaviour that is more regular and takes a value closer to 1. Weaker periodicity is closer to 0, indicating behaviour that is more random and irregular.

In a previous work, we have shown in human subjects how 24 h periodicity intensity correlates with the presence of blood markers which are known indicators of welfare [3]. While some work has been conducted in humans, there has been a limited extension of the investigation of periodicity in commercial calves, though a recent systematic review of commercial sensors in precision livestock farming found a range of sensor technologies including accelerometers, cameras, load cells, milk sensors, and boluses [4]. These are typically used to determine animal activity [5], feeding and drinking behaviour [6], and overall welfare [7]. There has been very little use of wearable sensors in calf welfare, especially in newborn calves, where the aim is to have high calf vitality, which can be defined as having the “capacity to live and grow with physical and mental energy and strength” [8]. This is despite the knowledge that for dairy cows, the consistency of behavioural patterns as measured by circadian rhythms is a characteristic of good health and welfare while changes to the regular patterns can be precursors of health issues [9,10].

For the research reported herein, we investigate the circadian periodicity of newborn calves and how the strength of that periodicity changes throughout their first 6–8 weeks. Our method generates a historical overview of each calf and this analysis is useful to examine the development and welfare of each individual calf. It is also possible that the regularity of the 24 h circadian rhythm could be a result of a herd effect, which is the focus herein. Many animals including cattle are behaviourally synchronised with herds, coordinating reactions to external zeitgebers [11]. If we determine a welfare indicator such as the periodicity intensity of the circadian rhythm, and apply this to individual calves in a herd, then the deviations of an individual calf from the herd should stand out, thus allowing us to identify calves whose individual welfare is poorer than that of the rest of the herd.

## 2. Materials and Methods

**Figure 1:**
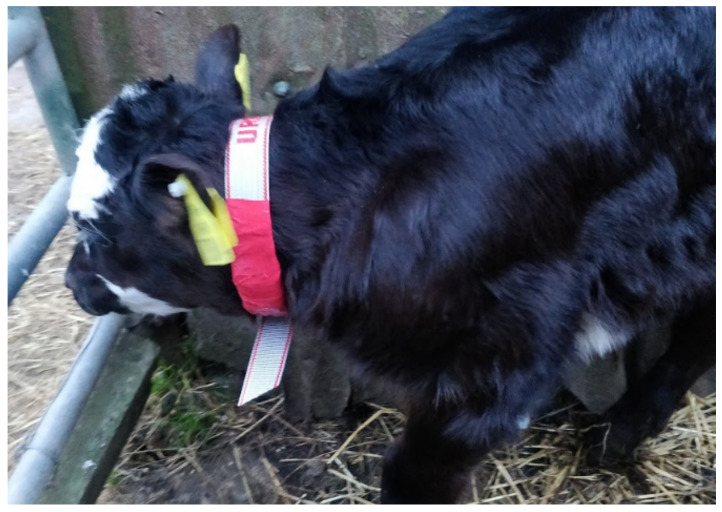
Calf wearing a collar with Axivity AX3 sensor securely affixed.

Data were gathered from 24 dairy calves, each monitored for 60 days from birth, between April and June 2019. The calves were spring-born at a commercial dairy farm. They were fed twice a day on bucket and teat, were bedded daily and had human contact during these activities. Feeding times would have been at similar times to milking as they were fed whole milk after the automated milk feeders had some technical issues. The wearable sensors used to collect data were Axivity AX3 sensors secured to a collar and placed around the necks of the newborn calves, as shown in Figure 1. The collars were applied between 1 and 5 days after the calves’ births.

The Axivity AX3 (Axivity, York, UK https://axivity.com/ (accessed on 15 June 2022)) is a popular data logger with a built-in micro-electromechanical system sensor in the form of a three-axis accelerometer, which enables it to monitor and record movement levels against a real-time clock. The expected battery life is 60 days between charges when using our parameters which were a sample rate of 12.5 Hz with 16-bit resolution and a range of + or − 8 G. As noted by Bucklet et al. [12], each sensor costs approximately EUR 120, measures 23.0 mm × 32.5 mm × 7.6 mm, weighs 11 g and has 512 MB of on-board memory for data storage. Data from the sensors can be accessed using the Open Movement (OmGUI) configuration and the analysis tool (https://github.com/digitalinteraction/openmovement/wiki/AX3-GUI/ (accessed on 15 June 2022)), an open source application to set up, configure, download and visualise data from Axivity sensors. Although they are waterproof to the IPx8 standard, they were wrapped in clingfilm, covered in masking tape and protective mesh and secured on the collar. By the end of the collection period, each sensor’s battery had run out but our goal was to collect data for at least 6 weeks and that was achieved.

Pre-processing data involved sampling x, y and z axis values into signal vector magnitude (SVM), a time-series independent of the sensor orientation and thus invariant to any movement of the collar around the neck, as we were interested in calves’ overall movement rather than movement in a particular direction. SVM shown below, also known as *Amag* [13], is a useful metric for calculating movements which are not axis-specific and is used extensively in pre-processing raw accelerometer data [14].

$$SVM=\sqrt{x^{2}+y^{2}+z^{2}}$$

A Butterworth fourth-order band-pass filter [15] with frequency in the range 0.5 Hz and 20 Hz was then applied to remove any white noise, and negative values were converted into absolute values. The final movement value used for analysing periodicity is the aggregated mean of SVM calculated over 60 epochs (seconds) which was chosen after we inspected different epochs between 1 min and up to 3600 s.

**Figure 2:**
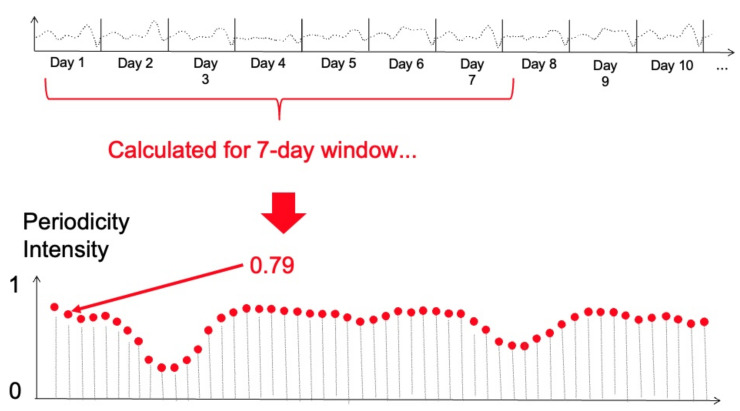
Schematic for the calculation of periodicity intensity for a calf. Figure shows 10 days of SVM values (top part of the figure) with a 24 h periodicity intensity for a 7-day window calculated starting just after the start of recorded data and yielding a value of 0.79. This sliding window will shift forwards in time and to the right on the schematic graph by 15 min and re-calculate the 24 h periodicity intensity. This is repeated until the end of the data recording gives the time series plotted on the bottom half of the Figure, with periodicity intensity values calculated at 15 min intervals.

Determining the intensity of 24 h periodicity over a time series of up to 8 weeks would result in a single periodicity intensity for weeks of data, which would not provide any insights into the behaviour during those weeks. Therefore, we used a sliding window of 7 days duration and determined the 24 h periodicity intensity for those 7 days, and then shifted it by 15 min before repeating the process across the next 7 days of data [16]. This time-lagged overlapping window across the entire data set for each calf provides intensity scores as schematically shown in Figure 2. The calculation of periodicity intensity over time was calculated for each calf.

To look for synchronous impacts on periodicity intensities across the herd of 24 calves at the same points in time, we aligned the time series of 24 h periodicity intensity values for each calf by date and time and we present this as stacked line graphs. In a stacked line graph, the x axis represents time from the beginning to the end of the logging period, while the y axis is a stack of periodicity intensities for each of the calves, each calf shown in a different colour. The top line of the overall graphs show the aggregated periodicities from all the calves and this will rise and fall during the logging period. Features to look for in the stacked line graph are where the overall graph rises or falls and where each or even many of the individual calf entries in the graph also rise or fall together; thus, the rises or falls in the overall graph can be attributed to many rather than a small subset of the calves’ periodicity intensities.

## 3. Results

**Figure 3:**
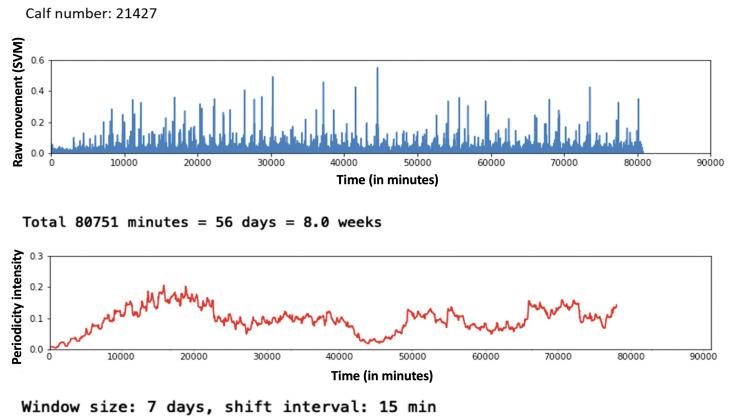
Raw data and periodicity intensity for calf 21427. The top graph shows the SVM values computed from raw x–y–z accelerometer data as one value per minute. Peaks throughout the logging period correspond to calf activity during the day and examining raw values of activity levels suggests a reasonably regular circadian rhythm for this calf. The bottom graph shows a time series of 24 h periodicity intensities, each value calculated from a 7-day period and the calculation is repeated every 15 min. This graph reveals a completely different picture for calf 21477 with a gradual acclimatisation to regular circadian rhythms for the first 14 days (c 19,000 min) followed by a drop-off reaching a low at approximately 31 days (c. 45,000 min) and then gradually recovering.

We analysed the 24 h periodicity intensities for the 24 calves to determine whether there were simultaneous changes observed across the herd. The development of periodicity intensity for one of the calves (number 21427) is shown in Figure 3. Periodicity intensity is generally weak for this calf, averaging at approximately 0.1 throughout the logging period, whereas most of the other calves had higher values. The explanation for this is that this calf is not as healthy as others in the herd. The periodicity intensity increased from when the sensor was affixed shortly after birth until 12–14 days (c. 19,000 min) of age after which there was a gradual drop-off with a low at approximately 31 days (c. 45,000 min) followed by a gradual recovery. This pattern is not visible in the raw SVM data shown in the top graph in Figure 3, and while we do not know the cause of this poor state of welfare at approximately day 31, it is useful to know whether this was systematic across the herd or specific to this particular calf.

Although the 24 calves had different dates of birth, all calves in the herd were contributing accelerometer data by 5 April. Analysing data from this date onwards enables us to combine data from older and younger calves. By this date, 5 calves were already 5 weeks old (born on 2 March), 4 were already about 3 weeks old (those born 14 March), 4 more were 2 weeks old (born on 21 March) and 11 were newborns (born on 5 April).

**Figure 4:**
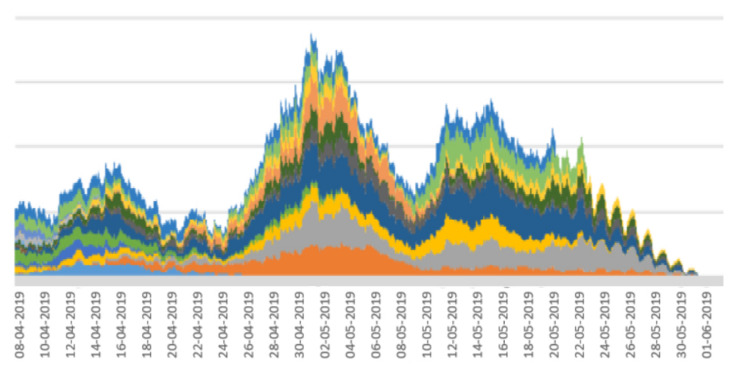
Stacked line graph for aligned periodicity intensities for 19 contributing calves. Each colour represents a different calf.

Among the 24 calves, 5 were outliers and were eliminated from further analysis as they had a large amount missing data due to their collars falling off and not being re-attached quickly enough. Such gaps in the recording of movement for an individual calf would disrupt the calculation of the shifting 7-day windows of periodicity intensity. The 19 remaining calves were used to generate the stacked line graph shown in Figure 4, where each colour represents a different calf and the analysis is applied to more than 1100 days of data gathering. The x axis in this figure represents dates. End dates are not consistent across individual calves due to the fact that either a sensor’s battery ran out or movement sensors with working batteries were removed from calves at different times following the data collection period.

In the analysis of the herd of calves in Figure 4, we see that periodicity intensity for the whole herd increased rapidly beginning on 24 April, rising up steadily until about 3 May. Directly following this, there was a clear drop in 24 h periodicity intensity across the herd, indicating a synchronous dis-improvement in welfare, which reached its low point after about 1 week, around 9 May. This was followed by a return to better welfare, although not as high as at 2 May, and at around 24 May, individual calf data collection started to end as the accelerometers were removed from calves or the battery ran out. Since periodicity intensity values are based on a 7-day sliding window, there will be a lag of some days between a stressful event occurring at a single point in time which could have caused this dis-improvement and evidence of it manifesting in the 7-day periodicity intensity window values. Thus, the drop-off, which reached its minimum point around 9 May, was most likely caused by a stressful or traumatic event at around 2 May.

**Figure 5:**
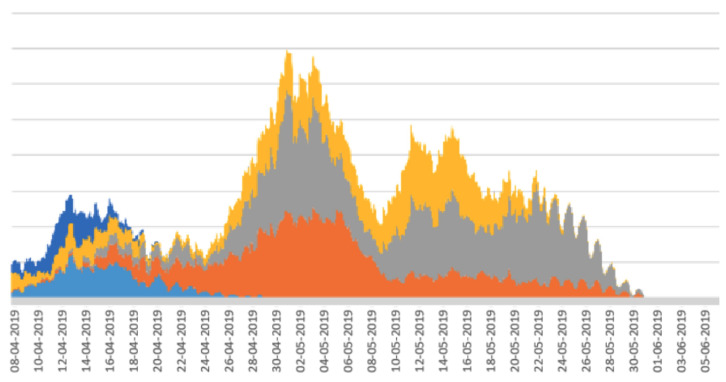
Stacked graph for aligned periodicity intensities for calves 1–5. Each colour represents a different calf.

**Figure 6:**
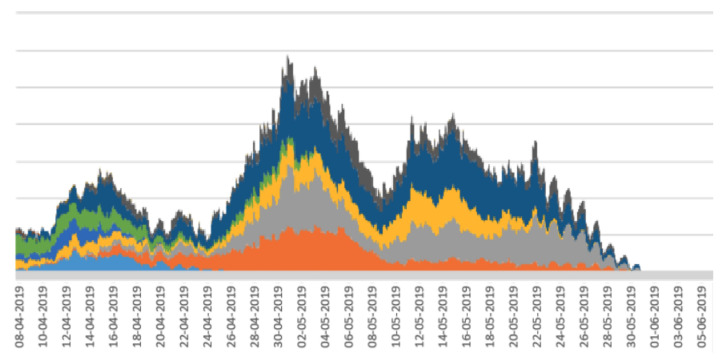
Stacked line graph for aligned periodicity intensities for calves 1–10. Each colour represents a different calf.

**Figure 7:**
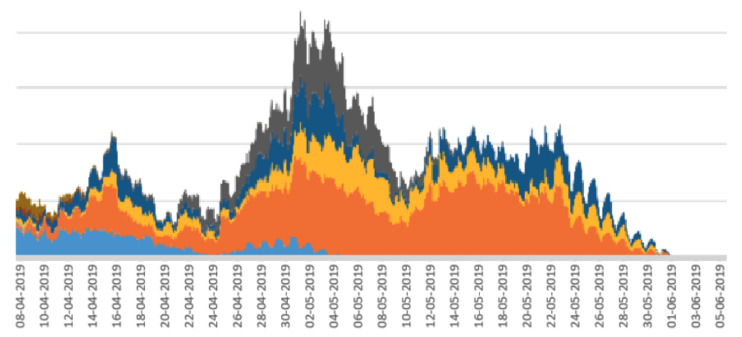
Stacked line graph for aligned periodicity intensities for calves 6–15. Each colour represents a different calf.

**Figure 8:**
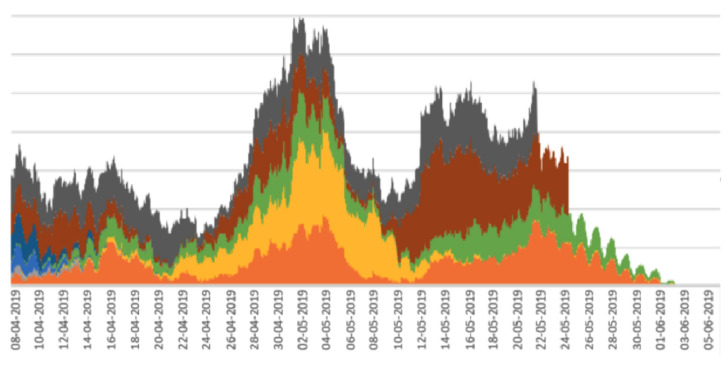
Stacked line graph for aligned periodicity intensities for calves 11–19. Each colour represents a different calf.

**Figure 9:**
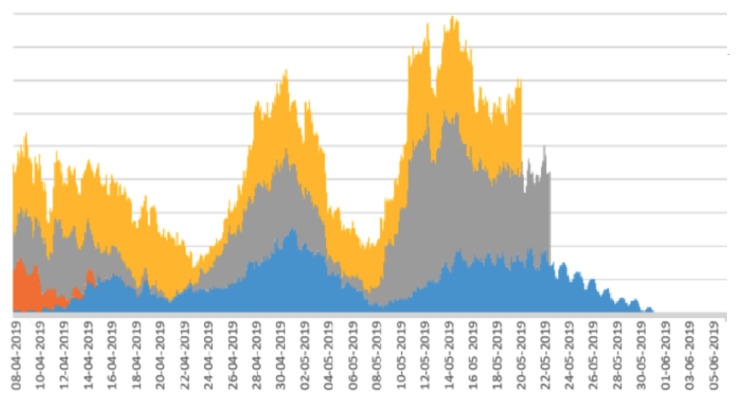
Stacked line graph for aligned periodicity intensities for calves 16–19. Each colour represents a different calf.

Stacked line graphs are useful for visualising the impact of some event across a set of variables. In the case of the work reported here, the variables are a set of periodicity intensity values. As shown in [17], stacked line graphs are known to present an illusion of peaks and troughs which might be caused by only a subset of the subjects, calves in our case, rather than across all of the subjects. This means that the overall stacked line graph might be artificially boosted or deflated by peaks or troughs from a small number of calves. To assess this, we divided the main from 19 calves into random sub-groups and plotted a stacked line graph for each sub-group. The rationale for this is that if the stacked line graphs for the sub-groups show the same shape as the stacked line graph for the whole herd, then there are no artificial boosts or troughs from a sub-group, and the pattern is more or less applicable to all calves in the herd. The stacked line graphs for the sub-groups are shown in Figure 5, Figure 6, Figure 7, Figure 8 and Figure 9.

The graphs in Figure 5, Figure 6, Figure 7, Figure 8 and Figure 9 show a remarkable consistency in their overall shape, thus illustrating that the majority of calves show synchronous upwards and downwards trends in their periodicity intensities. These illustrate that the peaks and troughs are not artificially boosted by the peaks and troughs of a sub-group of calves. With the sub-groups and the whole herd, the same pattern of three peaks is seen as in Figure 4 with the middle peak of the periodicity intensity as the highest also observed in each sub-group. From this, we infer that there were likely to be external events that affected the herd as a whole. While we were unable to determine the cause of this impact, it does suggest that periodicity intensity measurements are useful as indicators of the management of environmental factors that may impact on the welfare of calves. In the case of calf 21427 shown earlier in Figure 3, this calf’s periodicity intensity values do not precisely match the values of the overall herd, although the first and the third peak are present and distinguishable.

## 4. Conclusions

In this paper, we analysed three-axis accelerometer movement data sampled at 12.5 Hz from 19 calves over a period of approximately 8 weeks from birth. The magnitude of calf movement in 1 min epochs in any direction was calculated and used to compute and visualise the intensity of the 24 h periodicity at 15 min intervals as the calves became older.

We observe that the intensity of the 24 h periodicities for individual calves increase and decrease synchronously in a herd of 19 calves. Such patterns are not visible from the raw movement data and only by processing the data in this way are these insights revealed. Our confidence in this form of analysis is based on generating the visualisation from randomly selected sub-groups of calves as well as from all calves in the herd for which movement data for the full logging period is available.

The results demonstrate that there are factors which affect the welfare of the herd as a whole as calf welfare, for which periodicity intensity is an indicator, which rises and falls synchronously. Possible uses of this form of analysis would be the processing of movement or any other form of activity data in pseudo-real time in order to detect early stage changes in individual calves compared to changes in the rest of the herd. Thus, as the herd reacts to external stimuli and influences, both positive influences such as regular feeding or traumatic influences such as disbudding, an individual calf’s deviations from those synchronised behaviour changes by the herd can be detected. Our analysis appears to be more sensitive to overall herd and individual calf welfare than currently used forms of analysis which are based on movement data alone and are used to classify animal activity [5] including feeding and drinking [6], and overall welfare [7].

For future work, we recommend that a larger herd size be used for data gathering with a more detailed recording of the internal and external factors which might cause changes in calf or herd welfare such as weaning, turning out to pasture, disbudding, or even changes in weather.

## Data Availability

The data presented in this study are openly available in Figshare at https://doi.org/10.6084/m9.figshare.20039486 (accessed on 9 June 2022).

## Abbreviations

The following abbreviations are used in this manuscript:

PSD	Power Spectral Density
SVM	Signal Vector Magnitude
